# Systemic and Local Immunological Markers in Preeclampsia

**DOI:** 10.3390/diagnostics15131644

**Published:** 2025-06-27

**Authors:** Almagul Kurmanova, Altynay Nurmakova, Damilya Salimbayeva, Gulfiruz Urazbayeva, Gaukhar Kurmanova, Natalya Kravtsova, Zhanar Kypshakbayeva, Madina Khalmirzaeva

**Affiliations:** 1 Department of Obstetrics and Gynecology, Al-Farabi Kazakh National University, 71 Al-Farabi Ave., 050040 Almaty, Kazakhstan; gaukhar.kurmanova@kaznu.kz (G.K.); kypshakbaevazhanar@gmail.com (Z.K.); madinakhalmirzaeva7@gmail.com (M.K.); 2 Department of Strategic Development and Science, Scientific Center for Obstetrics, Gynecology and Perinatology, 125 Dostyk Ave., 050010 Almaty, Kazakhstan; sdamilya@mail.ru (D.S.); gulffa@mail.ru (G.U.); nat_kravtsova@mail.ru (N.K.); 3 Department of Obstetrics and Gynecology with a Course in Clinical Genetics, S.D. Asfendiyarov Kazakh National Medical University, 94 Tole bi Ave., 050012 Almaty, Kazakhstan

**Keywords:** pre-eclampsia, immune system, placenta, maternal medicine, cytokines

## Abstract

Preeclampsia (PE) is one of the main causes of obstetric complications and leads to both maternal and neonatal mortality. The maternal innate immune system plays an important role throughout pregnancy by providing protection against pathogens, while simultaneously inducing tolerance to a semi-allogenic developing fetus and placental development. **Background/Objectives**: To conduct a comparative study of immunological markers in the blood and placenta in preeclampsia. **Methods**: A total of 35 pregnant women were enrolled in a comparative study with preeclampsia (7) and with physiological pregnancy (28). A study of the immune status in peripheral blood and placenta was conducted with an examination of the subpopulation of lymphocytes profile and intracellular cytokines production by flow cytometry. **Results**: In the blood of pregnant women with PE, there was a decrease in CD14+ monocytes, as well as a significant increase of natural killers CD16+, CD56+ and activation markers HLA-DR+ and CD95+, as well as a significant rise in production of IL-10, TNF, Perforin, GM-CSF, and IGF. At the same time, in placental tissue in patients with preeclampsia, on the contrary, a significant decrease in regulatory cells CD4+, CD8+, CD14+, CD56+, CD59+, activation markers CD95+, as well as anti-inflammatory cytokine IL-10, growth factors VEGFR and IGF was detected. **Conclusions**: The maternal–fetal immune profile is crucial for successful fetal development and dysregulation of T-, B-, and NK cells can contribute to inflammation, oxidative stress, and the development of preeclampsia.

## 1. Introduction

Preeclampsia (PE) is one of the main causes of obstetric complications that increase both maternal and neonatal mortality [1]. In Kazakhstan, preeclampsia is second after extragenital diseases in the structure of causes of critical situations in obstetrics. The clinical picture of preeclampsia is characterized by elevated blood pressure of 140/90 mmHg after 20 weeks of gestation and proteinuria above 0.3 g/day [2]. However, in the absence of proteinuria, there may be concomitant symptoms such as edema, migraines, visual impairment, and other organ damage [2]. The etiology of preeclampsia is still unclear, but preeclampsia is nowadays divided into two phenotypes: early-onset and late-onset [3]. Up to 95% of cases are late-onset PE, while early-onset pre-eclampsia is associated with neonatal mortality and maternal morbidity [4]. Impaired placental development in early pregnancy and subsequent stunting are often associated with early-onset PE, while late-onset PE is thought to be associated with maternal endothelial dysfunction [5]. Both phenotypes result in an increased inflammatory response leading to adverse maternal and fetal complications. The main problem with early diagnosis of pre-eclampsia is that it is asymptomatic in the early stages [6].

The maternal innate immune system plays an important role throughout pregnancy in providing protection against pathogens, while simultaneously inducing tolerance to a semi-allogenic developing fetus and placental development. This is achieved through a precise balance of various cellular functions and timely interaction between cells of the innate immune system and other cells of the placenta/uterus [7]. This interaction is at the interface between mother and fetus in early pregnancy, which is important for the progression of pregnancy, namely, appropriate remodeling of the spiral arteries into low resistance, high-capacity vessels, coordinated with proper trophoblast invasion [8]. Natural killer (NK) cells can stimulate fetal growth and regulate delivery [9]. These decidual NK cells (dNKs) represent a population that differs both phenotypically and functionally from peripheral NK cells (pNKs). In contrast to pNKs, the dNK subpopulation has a CD56+/CD16− phenotype [10] and demonstrates a lower cytotoxic potential and a higher secretory cytokine profile. Decidual NK cells, secreting VEGF and IGF, stimulate remodeling of the spiral uterine artery, a process that is critical for successful formation of the placenta and the fetal–maternal interface during pregnancy [11]. While some studies have reported significantly lower numbers of NK CD56+ cells in decidua in preeclampsia [12], other reports have indicated the opposite trend [13]. The heterogeneity between studies and the differences in patient characteristics suggest possible explanations for these discrepancies. A recent study showed that the increase in dNK and pNK cells was higher in EOPE compared to LOPE [13].

Simultaneous analysis of both systemic and local immune compartments may offer more comprehensive insight into the immunopathogenesis of PE. In particular, immune cell profiling in placental tissue—where maternal–fetal immune interactions are concentrated—is rarely performed in parallel with peripheral blood assessments in the same cohort.

To our knowledge, few studies to date have employed flow cytometry to compare immune cell phenotypes in both blood and placental tissue from the same patients with late-onset PE. This dual-compartmental approach enables the identification of tissue-specific immune alterations and improves our understanding of immune dysregulation in PE.

Among the representatives of the colony stimulation factor (CSF) family, granulocyte-monocyte (GM)-CSF affects the survival and growth of myeloid leukocytes. There are conflicting reports as to whether GM-CSF levels are elevated in maternal and placental blood serum in patients with preeclampsia. A recent study indicates a reduced level of GM-CSF in preeclampsia compared to controls [14].

Effector CD8+ T cells play a crucial role in ensuring a balance between the tolerance of the fetoplacental unit and involvement in the immune response to infection. However, they are also thought to be involved in immune tolerance, which is crucial for a normal pregnancy [15]. It was found that the proportion of CD8+ memory cells was reduced during pregnancy with PE compared with the healthy group, but without data on the presence of cytotoxic proteins [16].

The main challenge in the analysis of immune cells during pregnancy is their dynamic fluctuations. As their number fluctuates during pregnancy [17], a suitable control group adapted to the gestational age is essential for the accurate interpretation of the results.

## 2. Materials and Methods

### 2.1. Subjects

Peripheral blood was analyzed in 35 women (7 from the preeclampsia group and 28 from the control) and placental tissue from the central part of the maternal side from 10 women (5—preeclampsia and 5—control).

*The criteria for inclusion in the PE group* were as follows: pregnant woman aged 18 years and over, gestational hypertension, increased blood pressure > 140/90 mmHg after 20 weeks, proteinuria > 0.3 g/day, as well as the presence of anamnestic, laboratory, and instrumental indications and clinical manifestations of organ lesions on the background of hypertension.

*Criteria for inclusion in the control group*: pregnant woman over the age of 18, pregnancy is not complicated by hypertension.

*Exclusion criteria from both groups*: acute and chronic inflammatory diseases, severe extragenital pathology, history of organ transplantation, history of cancer, diabetes mellitus, blood transfusion, systemic autoimmune diseases, tuberculosis, HIV, chronic arterial hypertension. All patients signed an informed consent to participate in the study.

### 2.2. Ethics Approval

The work was carried out in accordance with the principles of voluntariness and confidentiality based on the Code of the Republic of Kazakhstan “On the Health of the People and the Healthcare system” (dated 7 July 2020, No. 360-VI SAM) and with the Helsinki Declaration. The study was approved by the Local Ethical Committee of Scientific Center of Obstetrics, Gynecology and Perinatology (No. 2 dated 9 November 2022). All participants provided written informed consent for the use of biomaterials in this study.

Due to ethical constraints and the difficulty of obtaining high-quality placental samples immediately after delivery, the number of participants included for placental tissue analysis was limited to five in each group. This sample size, while sufficient for preliminary observations, may reduce the statistical power of the findings and should be interpreted accordingly.

### 2.3. Sample Preparation

The venous blood/placenta was collected immediately after the delivery of the women and transferred to the laboratory of the Scientific Center for Obstetrics, Gynecology and Perinatology (Almaty, Kazakhstan).

*Venous Blood Sample Preparation.* Venous blood (5 mL) was collected from each participant into tubes containing EDTA. The blood was conducted according to the manufacturer’s protocol (https://www.bdbiosciences.com/en-us/resources/protocols, accessed on 8 June 2025). Briefly, the blood was treated with a protein transport inhibitor (contains monensin) BD GolgiStop™, resuspended and transferred to plastic tubes for immunofluorescent staining. For surface marker analysis, 50 µL of the blood sample was incubated with 5 µL of fluorochrome-conjugated monoclonal antibodies (mAb) for 15 min at room temperature in the dark. After staining, erythrocytes were lysed using BD FACS™ Lysing Solution, incubated for 10 min in the dark at room temperature, and centrifuged at 300× *g* for 5 min. The supernatant was removed, resuspended cells were processed with Cytofix/Cytoperm™ Plus Fixation/Permeabilization Kit, followed by mAb for staining and binding intracellular receptors.

*Placental Tissue Preparation*. Placental tissue samples (2 × 2 cm) were obtained from the central maternal side and placed in sterile tubes containing phosphate-buffered saline. The samples were mechanically dissociated using Medicon containers and a Medimachine homogenizer (BD, San Diego, CA, USA). The resulting cell suspension was centrifuged over a Ficoll–Verografin density gradient (d = 1.078 g/mL) for 30 min to isolate mononuclear cells. Placental leukocytes were processed according to the manufacturer’s protocol (https://www.bdbiosciences.com/en-us/resources/protocols, accessed on 8 June 2025). Briefly, placental leukocytes were treated with a protein transport inhibitor (contains monensin) BD GolgiStop™, after staining surface receptors with fluorochrome-conjugated mAb, permeabilized with Cytofix/Cytoperm™ Plus Fixation/Permeabilization Kit, resuspended cells were processed by mAb for staining and binding intracellular receptors.

Surface markers: CD4 FITC (clone RPA-T4), CD8 FITC (RPA-T8), CD14 FITC (M5E2), CD16 FITC (3G8), CD56 FITC (B159), CD19 FITC (HIB19), CD59 PE (p282 (H19)), CD95 PE (DX2), HLA-DR PerCP (L243).

Intracellular markers: TNF PerCP-Cy5.5 (Mab11), IL-10 PE (JES3-19F1), GM-CSF PE (BVD2-21C11), VEGFR-2 PE (CD309, clone 89106), IGF PE (CD221, clone 1H7), Perforin PerCP-Cy5.5 (clone δG9) (all from BD Biosciences, USA).

### 2.4. Flow Cytometric Analysis

First, the total population of leukocyte cells was isolated using the CD45+ marker, then lymphocytes and monocytes were isolated from this fraction. For proper flow cytometry analysis, cells were examined using light microscopy to confirm that they are well dispersed. Concentration-matched isotype controls were used to set the gates and single-fluorochrome stained controls were used to compensate for spectral overlap. The triple-color immunophenotyping of cells was evaluated on a BD FACS CALIBUR flow cytometer (USA) and the data was analyzed using the CELLQuest program.

The results of immunology included the following indicators: CD4+ (helper T cells)—play a key role in activating the immune system, CD8+ (cytotoxic T cells)—are necessary for the destruction of infected or tumor cells. CD19+ is a marker of B-lymphocytes, which produce antibodies during a humoral immune response. HLA-DR+ is a marker of immune response activation, is a molecule of the main histocompatibility complex class 2, and plays an important role in the presentation of antigens to T cells. CD16+, a receptor that is expressed on the surface of NK cells, plays a key role in opsonization and destruction of antibody-dependent cells. CD56+ is an NK cell marker that plays an important role in innate immunity, protecting the body from viral infections and tumor cells. CD59+ is a membrane protein that inhibits complement activation, protecting the body’s own cells from lysis. IL-10 is a cytokine that is an important regulator of the immune response and has anti–inflammatory properties. TNF-α (tumor necrosis factor) is a cytokine that regulates the inflammatory process and the immune response. GM-CSF is a cytokine that stimulates the production of granulocytes and macrophages, thereby supporting and activating cells of the immune system. VEGF2 is a growth factor that promotes angiogenesis. Perforin is a protein that is secreted by cytotoxic cells and forms pores in the membrane for target cells, helping to enter the cell and cause apoptosis.

### 2.5. Statistical Analysis

Statistical calculations and analysis were performed in the Jamovi program [2]. To compare the characteristics of the studied groups, due to the small sample, the Fisher criterion was used (for nominal data). The statistical significance of the differences in quantitative data between the groups was calculated using the Mann–Whitney U-test. Confidence intervals (CI) were calculated for key indicators. *p*-values < 0.05 were considered statistically significant. To assess the degree of differences between the groups, in addition to the standard check of statistical significance (*p*-values), the study used effect size indicators. Cohen’s effect size (Cohen’s h) was used in the analysis of dichotomous (categorical) variables. This indicator makes it possible to estimate the difference between the two proportions, giving an idea of the scale of differences between the groups, even if statistical significance is not achieved with a small number of observations. The Cliff’s delta effect size was used in the analysis of nonparametric quantitative data. Cliff’s delta estimates the probability that a randomly selected value from one group will be higher or lower than the value from the other group.

## 3. Results

### 3.1. Clinical and Pathological Characteristics of Studied Groups

Clinical and pathological characteristics of studied groups are presented in Table 1, Table 2, Table 3 and Table 4.

In women with preeclampsia, blood pressure was significantly higher (*p* < 0.05), and the gestational age at the time of delivery was shorter (*p* < 0.05) compared to the control group. The weight of the child was significantly lower in women with preeclampsia, which may indicate fetoplacental insufficiency in preeclampsia. Most women in both groups were overweight.

There was a statistical difference between acetylsalicylic acid (100%) and methyldopa (85.7%) taken by women with preeclampsia, compared to the control group, where 53.6% took acetylsalicylic acid and no methyldopa was prescribed to anyone (*p* < 0.05). An interesting observation was the significantly lower use of iron-containing medications in the PE group (*p* < 0.05). This may be due either to less pronounced iron deficiency anemia in patients with PE, or to restrictions on taking medications against the background of hypertension or other obstetric risk. This aspect requires further analysis, including taking into account ferritin and hemoglobin levels.

There was also a trend towards a significant difference in the incidence of thyroid diseases (*p* = 0.079), as well as a burdened family history of PE (*p* = 0.095), which may indicate the possible involvement of endocrine factors and genetic predisposition in the pathogenesis of the disease. These observations correspond to a number of literature data on the role of thyroid dysfunction and hereditary factors in the development of preeclampsia.

Thus, the data obtained make it possible to identify a number of potential risk factors and clinically significant markers that may be useful for more accurate risk stratification of PE, especially in the early stages of gestation.

In this study, Cohen’s h effect size was calculated to assess differences in the frequency of clinical and anamnestic signs between groups of women with preeclampsia and healthy pregnant women. This indicator makes it possible to complement the traditional statistical analysis with an assessment of the clinical significance of the identified differences.

The most significant differences were found in parameters such as the use of methyldopa (Cohen’s h = 2.77) and acetylsalicylic acid (h = 1.37). These values indicate an extremely high degree of differences between the groups, which confirms the specific purpose of these medications exclusively for preeclampsia or a high risk of its development. In addition, for iron-containing drugs, the effect size was also significant (h = 0.98), and iron preparations were more often taken by women in the control group. This may reflect a more active prevention of anemia in the group without complicated pregnancy.

Moderate effects (h ≈ 0.5–0.6) were observed in the incidence of thyroid diseases and varicose veins; however, the statistical significance of these differences was not achieved, probably due to the limited sample size.

The differences between the groups were statistically significant (*p* < 0.05). In the preeclampsia group, emergency caesarean section was more common, while in the control group, vaginal delivery (spontaneous and induced) was more common. In the control group, there were non-medical indications for induced labor and caesarean section (the woman’s desire).

A general blood test of women with preeclampsia showed a reduced level of hemoglobin, which may indicate anemia. A general urine analysis indicated elevated protein in the urine, which indicates proteinuria.

### 3.2. Immunological Parameters in Peripheral Blood

Immunological parameters in peripheral blood are shown in Table 5.

In the preeclampsia group, the statistically most significant differences were found for the following markers in peripheral blood: increased levels of CD16+, CD56+, HLA-DR+, CD95+, and intracellular production of cytokines—IL-10, TNF, Perforin, GM-CSF, IGF, while reducing CD14+ levels.

The immune profile of peripheral blood in women with preeclampsia shows marked changes in both cellular composition and intracellular expression of key inflammatory and angiogenic markers. One of the most significant differences was a strong increase in the expression of the CD95+ apoptosis receptor (Fas) in the PE group compared with the control (72.3 ± 11.9 vs. 10.4 ± 1.72; *p* < 0.001), which may indicate the active initiation of apoptotic processes characteristic of chronic hypoxia and systemic inflammation. Activation of antigen-presenting cells is confirmed by an increase in the level of HLA-DR+ (24.8 ± 4.36 versus 1.48 ± 0.468; *p* < 0.001), as well as a decrease in CD14+ monocytes (2.32 ± 0.85 in PE versus 63.4 ± 4.16 in control; *p* < 0.001), reflecting the involvement of the innate immune link in pathogenesis.

It was interesting to note the increase in cytotoxic markers such as Perforin (62.5 ± 6.33 vs. 15.2 ± 4.30; *p* < 0.001), which indicates the activation of CD8+ T cells and/or NK cells potentially involved in the destruction of placental tissue. The increased TNF+ level (13.3 ± 3.26 vs. 1.09 ± 0.482; *p* < 0.001) also highlights the pronounced pro-inflammatory status.

An important point is a significant decrease in VEGF2 level in PE (9.01 ± 1.24 versus 21.6 ± 1.47; *p* < 0.001), which corresponds to a violation of vascular remodeling and angiogenesis, a key link in the pathogenesis of the disease. At the same time, the level of IGF is significantly higher in the PE group (78.6 ± 6.73 vs. 8.84 ± 1.49; *p* < 0.001), which may be a compensatory mechanism aimed at stimulating the vascular network.

An increase in IL-10 (2.60 ± 1.15 vs. 1.72 ± 0.63; *p* < 0.05), despite the general increase in pro-inflammatory reactions, may be an attempt by the body to compensate for inflammation through regulatory mechanisms.

Using Cliff’s delta made it possible to supplement the interpretation of *p*-values and objectively assess the scale of differences, especially in small sample conditions.

### 3.3. Immunological Parameters in Placental Tissue

To gain a deeper understanding of the pathogenesis of preeclampsia, in addition to peripheral blood analysis, an immune profile study was conducted in placental tissue. It is especially important to take into account that it is the local imbalance of proinflammatory and regulatory cytokines, as well as a violation of the angiogenic response in the placental zone, that can play a key role in the development of vascular pathology and systemic hypertension. The results of immune markers in placental tissue by flow cytometry are shown in Table 6.

The obtained flow cytometry data allow us to identify significant changes in the immune profile of placental tissue in preeclampsia compared with the normal course of pregnancy. The revealed differences affect both surface markers of lymphocytic subpopulations and intracellular signaling molecules, which indicates a violation of the immune balance at the local level.

One of the key observations was a significant decrease in the content of CD4+ T helper cells and CD8+ cytotoxic T cells in preeclampsia. This indicates a deficiency in the adaptive cellular immune response in the placenta, which may interfere with the normal immune interaction between the maternal body and the fetus. The decrease in CD4+ cells, which play a central role in regulating the immune response, is particularly noteworthy.

A significant decline in the number of CD56+ NK cells involved in spiral artery remodeling and trophoblast invasion is also noteworthy. Their deficiency in preeclampsia may be associated with impaired vascular remodeling, which confirms the statement of inadequate trophoblast invasion as one of the central mechanisms of the disease pathogenesis.

Against this background, there is an increase in the level of CD16+ cells, which are associated with pro-inflammatory activity and may indicate increased local inflammation in the placental tissue. This is confirmed by a decrease in the expression of IL-10, a key anti-inflammatory cytokine, which indicates a shift towards the Th1/Th17 profile and a decrease in the regulatory mechanisms of immune defense.

The revealed significant decrease in the level of VEGF2 cells confirms the involvement of impaired angiogenesis in the pathogenesis of preeclampsia. Given the role of VEGF in the formation and maintenance of the vascular network, such changes may be the cause of placental hypoperfusion characteristic of PE.

Additionally, a decrease in Perforin cells involved in cytolytic activity may indicate functional depletion of NK and cytotoxic T cells, which disrupts the processes of immune surveillance in the tissue.

Interestingly, the levels of perforin and HLA-DR+ did not differ statistically significantly between the groups, which may indicate the preservation of certain aspects of cytotoxic activity or a high variability in the expression of these markers in PE.

TNF expression was absent in placental tissues during preeclampsia, while it was detected in the control, which may be due to depletion of the pro-inflammatory signal against the background of chronic inflammation or apoptosis of TNF-producing cells.

Thus, the revealed immunological changes confirm the multifactorial nature of disorders in preeclampsia, including deficiency of regulatory and angiogenic factors, depletion of cells of adaptive and innate immunity, as well as activation of pro-inflammatory mechanisms.

## 4. Discussion

The main conclusions of this study are:

➢ The weight of a newborn born to a mother who had preeclampsia is significantly lower than that of mothers with a physiological pregnancy.
➢ Iron preparations were used more often in the PE group (71.4%) than in the control group (25%) (*p* < 0.05).
➢ Women with preeclampsia have a higher chance of undergoing an emergency caesarean section.
➢ A statistically significant difference in immunological parameters was found in the group with preeclampsia.

Our study revealed contrasting immune profiles in maternal blood and placental tissue. In peripheral blood, markers of immune activation and cytotoxicity (e.g., CD95+, GM-CSF, Perforin) were elevated in PE patients, while placental tissue exhibited overall immune suppression, including reduced expression of CD4+, CD8+, CD14+, CD16+, and CD56+ cells.

This divergence may reflect immune compartmentalization, where the systemic and local immune environments respond differently to pathological stimuli. The placental microenvironment, influenced by hypoxia and oxidative stress, may result in the local suppression or exhaustion of immunity, while the peripheral blood reflects a more activated immune state. The placenta exhibits a distinct immune microenvironment characterized by resident macrophages (Hofbauer cells), decidual NK cells, and trophoblast-mediated immunomodulation. In PE, placental macrophages shift toward pro-inflammatory M1 polarization (e.g., upregulation of NOS2, TLR4, and HLA-DR in early PE) [18], while peripheral blood monocytes/macrophages may exhibit a compensatory shift toward anti-inflammatory activity. This immune compartmentalization is enhanced by the placenta’s unique exposure to hypoxia and damage-associated molecular patterns (DAMPs) that induce local inflammation via TLR activation [19]. Conversely, peripheral immune cells are influenced by systemic antiangiogenic factors (e.g., sFlt-1) and circulating syncytiotrophoblastic debris that promote endothelial dysfunction and systemic inflammation.

The immunological profile during pregnancy is dynamic, and efficient spiral artery remodeling depends on an anti-inflammatory environment that ensures immune tolerance between mother and fetus via the secretion of TGF-β, IL-10 and arginase-1 [20]. In PE, pro-inflammatory macrophages and Th17 memory cells dominate in the placenta, which is mediated by IGF1-IGF1R signaling. In contrast, decreased Tregs and an increased Th1/Th17 ratio are observed in the peripheral blood, suggesting systemic immune activation. This divergence may stem from placental-specific factors like syncytiotrophoblast stress, which releases pro-inflammatory cytokines (e.g., IL-6, TNF-α) locally while triggering compensatory anti-inflammatory responses peripherally. This peripheral compensation is evidenced by a slight elevation in IL-10 in PE, suggesting the immune system’s attempt to mitigate inflammation [21]. In preeclampsia, there is an increase in the level of pro-inflammatory cytokines such as TNF, IL-6, and IL-17, which contributes to the development of cytotoxic inflammation [22]. This is consistent with our data, which show that TNF was elevated in the group of women with PE. In vitro studies showed that TNF macrophage secretion induced apoptosis in trophoblast cells, which may underlie reduced trophoblast invasion and the inadequate remodeling of the spiral artery observed in PE [23].

The function of NK cells is determined by the balance between signals received through their killer-activating receptors (KARs) and killer-inhibiting receptors (KIRs); additional signals from cytokine and CD16+ receptors further regulate NK cell activation [24]. After activation, NK cells degranulate and release lysosomes containing perforin and granzymes, which induce lysis of target cells. In addition, NK cells produce pro-inflammatory cytokines such as IFNγ and TNF, which promote the activation of neighboring immune cells [20]. However, CD16+ is not highly specific for natural killers and can be detected on the surface of monocytes and parts of dendritic cells. Currently, the generally accepted way to identify human NK cells is to determine CD3+/CD14+/CD19+ negative lymphocytes expressing CD56+ cell adhesion molecules. In our study, molecules such as CD56+ and CD14+ were considered for the first time in the context of complications in PE. There was the dysregulation of innate immune function, an increased level of CD56 and a decreased level of CD14+.

A recent study isolated HLA-DR from circulating extracellular vesicles, and found high levels of HLA-DR in blood plasma in women with PE [25]. Our findings align with these data, showing that a significant increase in the expression of activation markers (HLA-DR+) and apoptosis (CD95+) indicates the hyperactivation of immune cells and their subsequent death in PE. This is consistent with data on the hyperactivation of the complement system and an increase in the circulating products of its activation in severe forms of PE [26].

Increased levels of angiogenic factors such as VEGFR are noted in PE. An imbalance between these factors can lead to endothelial dysfunction and insufficient blood supply to the placenta [27,28]. However, researchers have reported decreased serum IGF and placental tissue levels in a PE group compared to a control group [29]. A study on mice showed that GM-CSF expression in PE-affected placentas was significantly lower compared to that in healthy pregnant mice [30]. Conversely, another mouse study reported increased levels of GM-CSF and macrophages in placental tissue [14]. The research conducted by Weel et al. indicated that there was no statistically significant difference between GM-CSF levels in PE and control groups [31]. However, in our study, we obtained data showing that GM-CSF increased expression in the PE group.

The increased expression of CD95 (Fas) and perforin in women with PE reflects increased apoptosis and cytotoxic immune activation aimed at eliminating activated or damaged immune cells, which contributes to immune homeostasis [18]. However, in preeclampsia, dysregulated CD95 expression may lead to excessive apoptosis or immune imbalance, exacerbating placental inflammation. Perforin, a pore-forming protein expressed by cytotoxic CD8+ T cells and NK cells, facilitates granzyme entry into target cells, inducing apoptosis rapidly upon immune activation [32]. Elevated perforin levels suggest increased cytotoxic potential, which may contribute to tissue remodeling or damage within the placenta. Importantly, these two pathways—CD95-mediated apoptosis and perforin-dependent cytotoxicity—can function synergistically or compensate for each other in immune regulation.

From a clinical perspective, altered CD95 and perforin expression profiles hold promise as predictive biomarkers for immune dysregulation in preeclampsia. For instance, changes in peripheral blood CD95/CD27 ratios and perforin-expressing lymphocyte subsets have been linked to disease severity and progression [33]. Furthermore, targeting these molecules offers potential therapeutic avenues: perforin inhibitors are being explored to modulate excessive cytotoxicity in autoimmune and inflammatory diseases, while CD95 agonists or antagonists could be used to, respectively, induce apoptosis in pathological cells or protect healthy tissues from immune-mediated damage [9]. Understanding the compartment-specific regulation of these pathways—particularly the balance between placental and systemic immune responses—will be critical for developing targeted interventions that minimize off-target effects.

Although we focused on the immunological profile of placental tissue in our study, it is important to consider structural and histopathological alterations in the placenta associated with preeclampsia. In research conducted by Zou G et al., placental tissue was examined in women with preeclampsia and healthy women. In pregnant women with preeclampsia, the pathoanatomic examination of placental tissues revealed trophoblast proliferation, enlarged syncytial nodules, extracellular matrix deposition around the villi, apparent fibrinoid necrosis of the villi, edema of the placental blood vessels and the narrowing of the lumen, the enlargement of the villi blood vessels, and the thickening of the vascular basement membrane. Extensive villous hemorrhages and placental infarctions were prominent in preeclamptic placentas. In contrast, in healthy pregnant women, the placental villi had a homogeneous stroma and an intact vascular structure, with only occasional hemorrhagic spots and fibrous necrosis of the villi [34].

The placental microenvironment, characterized by hypoxia, oxidative stress, and altered cytokine signaling, may suppress the infiltration or retention of immune cells. At the same time, systemic compensation may manifest as heightened peripheral immune activation in response to placental dysfunction. These findings highlight the importance of evaluating both compartments to understand the full immunopathological context of preeclampsia.

## 5. Limitations and Future Directions

One of the key limitations of this study was the small sample size, particularly for the placental tissue group (*n* = 5 per group). This constraint may affect the statistical robustness and generalizability of our findings. While our results highlight significant changes in the local immune profile associated with PE, further investigations involving larger patient cohorts, histopathological analysis, molecular profiling, and clinical data integration are necessary to comprehensively elucidate the complex pathophysiology of the disorder. Multidisciplinary approaches will improve the diagnostic and prognostic potential of immune biomarkers in placental tissue.

Another limitation of our study is the restricted antibody panel used for flow cytometric analysis, particularly regarding the identification of NK cells. We relied on CD56+ and CD16+ markers alone, without additional lineage exclusion markers such as CD3+, CD14+, or CD19+, which are typically used to accurately define NK cell subsets (e.g., CD3-CD56+ NK cells). As a result, some overlap with other CD16+ populations (e.g., monocytes or dendritic cells) cannot be excluded. This limits the precision of phenotypic classification and interpretation of certain immune cell populations. Future studies employing expanded antibody panels will be essential for fully characterizing immune cell subsets involved in PE.

## 6. Conclusions

Based on the analysis of lymphocyte subpopulations in peripheral blood and placental tissue, our analysis revealed significant immunological disorders in women with PE compared to the control group. In the blood of pregnant women with PE, CD14+ monocytes were decreased, while cytotoxic NK cell populations (CD16+ and CD56+) and activation markers (HLA-DR+ and CD95+) were significantly increased, indicating increased apoptotic activity of immune cells. There was also a sharp increase in the intracellular expression of IL-10, TNF, Perforin, GM-CSF, and IGF.

At the same time, in placental tissue in patients with preeclampsia, on the contrary, a significant decrease of regulatory cells CD4+, CD8+, CD14+, CD56+, CD59+, activation markers CD95+, as well as anti-inflammatory cytokine IL-10, growth factors VEGFR and IGF was detected. The observed decrease in immune regulatory factors in the placenta suggests impaired immunoregulation, leading to local inflammation and cytotoxic stress in the area of uteroplacental interaction.

Our findings highlight an inconsistency between the systemic (blood) and local (placenta) immune response, which may be a key pathogenetic mechanism in preeclampsia development. Increased local inflammation against the background of suppressed systemic immunity contributes to the disruption of trophoblast invasion, angiogenesis deficiency, and the progression of clinical manifestations of the disease.

## Figures and Tables

**Table 1:** Demographic and clinical-pathological characteristics of patients. Clinical data and pregnancy outcomes of the PE and control groups.

Indicators	PE (*n* = 7)	95% CI	Control (*n* = 28)	95% CI	*p*-Value
Age	32.7 ± 3.95	(26;37)	32.5 ± 6.48	(21;45)	0.923
Weight	82.1 ± 9.37	(66;92)	80.9 ± 15.3	(61;132)	0.843
Height	166 ± 1.5	(163;167)	164 ± 5.65	(153;175)	0.332
Blood pressure					
Systolic	139 ± 11	(130;160)	102 ± 9.57	(80;120)	<0.001
Diastolic	84.3 ± 7.87	(70; 90)	67.5 ± 12.4	(60;120)	0.002
Gestational age at diagnosis	36.2 ± 2.14	(34.1 ± 40.1)	-	-	
Gestation period	38 ± 1.48	(36.6;41.0)	39.4 ± 1.26	(37.4;41.3)	0.018
Child’s weight in g	3041 ± 643	(2170;3900)	3618 ± 401	(2680;4270)	0.006

**Table 2:** Demographic and clinical-pathological characteristics of patients. Anamnesis of the life of the group with PE and control.

Indicators	Yes|No	Preeclampsia (*n* = 7)	Control (*n* = 28)	*p*-Value	Cohen’s Effect Size
Sister or mother had a PE	yes	2	1	0.095	0.748
	no	5	27		
Allergy	yes	2	10	1	−0.153
	no	5	18		
COVID-19	Yes	3	5	0.312	0.555
	no	4	23		
Vaccination	yes	2	8	1	0
	no	5	20		
Anemia	yes	1	11	0.380	−0.58
	no	6	17		
Obesity	yes	3	8	0.652	0.3
	no	4	20		
Kidney diseases	yes	2	8	1	0
	no	5	20		
Liver diseases	yes	1	1	0.365	0.395
	no	6	27		
Thyroid diseases	yes	3	3	0.079	0.761
	no	4	25		
Varicose veins of the lower extremities	yes	1	10	0.392	−0.506
	no	6	18		
Acetylsalicylic acid	yes	7	15	<0.05	1.499
	no	0	13		
Methyldopa	yes	7	1	<0.001	2.761
	no	0	27		
Calcium preparations	yes	4	20	0.652	−0.3
	no	3	8		
Iron preparations	yes	2	21	<0.05	−0.967
	no	5	7		

**Table 3:** Demographic and clinical-pathological characteristics of patients. Methods of delivery in the preeclampsia and control group.

Method of Delivery	PE (*n* = 7)	Control (*n* = 28)
Spontaneous Vaginal (1)	0	8
Induced Vaginal (2)	3	11
Elective Caesarean (3)	0	8
Emergency Caesarean (4)	4	1

**Table 4:** Demographic and clinical-pathological characteristics of patients. Laboratory parameters of the PE group.

Indicators	PE
*General blood test*	
Hemoglobin (g/L)	108 ± 15.9
Red blood cells (×10^12^/L)	4.25 ± 0.5
White blood cells (×10^9^/L)	8.54 ± 2.29
Platelets (×10^9^/L)	251 ± 58.6
*General urine test*	
pH	acidic
Protein (g/L)	0.414 ± 0.442
Relative density	1018 ± 9.51
*Biochemical analysis*	
Bilirubin (mmol/L)	6.87 ± 3.5
Aspartate aminotransferase, AST (U/L, ME/L)	21.4 ± 9.97
Alanine aminotransferase, ALT(U/L, ME/L)	15.9 ± 11.9
Urea (mmol/L)	4.16 ± 2.05
Creatinine (mmol/L)	66.6 ± 15.6
Total protein (g/L)	67.1 ± 6.77
Glucose (mmol/L)	4.98 ± 0.557
Fibrinogen (g/L)	4.12 ± 0.433
Activated partial thromboplastin time (sec)	30.4 ± 2.81
Prothrombin time (s)	9.98 ± 4.20
International normalized ratio	0.953 ± 0.08
Quick Prothrombin time (%)	102 ± 6.79

**Table 5:** Immunological markers in peripheral blood in both groups.

Lymphocyte Subpopulations	PE (*n* = 7)	Control (*n* = 28)	*p*-Value	Cliff’s Delta Effect Size
Surface markers				
CD4+	37.2 ± 8.29	35.6 ± 5.54	0.548	0
CD8+	32.7 ± 9.04	34.1 ± 3.03	0.726	0
CD16+	14.1 ± 2.92	1.38 ± 0.426	<0.001	1
CD56+	22.7 ± 5.54	11.5 ± 1.54	<0.001	1
HLA-DR+	24.8 ± 4.36	1.48 ± 0.468	<0.001	1
CD95+	72.3 ± 11.9	10.4 ± 1.72	<0.001	1
CD14+	2.32 ± 0.85	63.4 ± 4.16	<0.001	−1
CD19+	12.1 ± 4.55	9.9 ± 1.07	0.523	0
CD59+	56.3 ± 17.7	59.5 ± 5.85	0.470	0
Intracellular markers				
IL-10	2.60 ± 1.15	1.72 ± 0.63	<0.05	1
TNF	13.3 ± 3.26	1.09 ± 0.482	<0.001	1
Perforin	62.5 ± 6.33	15.2 ± 4.30	<0.001	1
VEGFR2	21.9 ± 3.87	21.6 ± 1.74	0.710	0
GM-CSF	69.1 ± 2.84	9.01 ± 1.24	<0.001	1
IGF	78.6 ± 6.73	8.84 ± 1.49	<0.001	1

**Table 6:** Immunological markers in placental tissue in both groups.

Lymphocyte Subpopulations	PE (*n* = 5)	Control (*n* = 5)	*p*-Value	Cliff’s Delta Effect Size
Surface markers				
CD4+	10.1 ± 0.54	17.0 ± 2.42	<0.001	−1
CD8+	3.40 ± 1.87	7.74 ± 2.50	<0.05	−0.9
CD16+	26.5 ± 1.02	18.2 ± 1.74	<0.001	1
CD56+	0.80 ± 0.35	3.42 ± 0.973	<0.001	−1
HLA-DR+	15.3 ± 5.76	14.5 ± 10.2	0.859	0
CD95+	15.4 ± 1.32	18.3 ± 2.40	<0.05	−0.68
CD14+	2.10 ± 0.87	3.74 ± 1.35	<0.05	−0.92
CD19+	7.40 ± 0.605	10.4 ± 1.16	<0.001	−1
CD59+	0.7 ± 0.05	1.80 ± 0.490	<0.001	−1
Intracellular markers				
IL-10	2.10 ± 0.769	4.36 ± 1.33	<0.001	−1
TNF	0	1.09 ± 0.482	-	−1
Perforin	5.40 ± 1.67	8.08 ± 1.91	<0.05	−0.76
VEGFR2	0.2 ± 0.045	1.46 ± 0.383	<0.001	−1
GM-CSF	6.20 ± 2.84	5.58 ± 1.15	0.261	0.04
IGF	0.6 ± 0.332	1.72 ± 0.581	<0.05	−1

## Data Availability

The data presented in this study are available on request from the corresponding author. The data are not publicly available due to privacy and ethical issues.

## Abbreviations

The following abbreviations are used in this manuscript

PE	Preeclampsia
dNK	Decidual Natural Killer cells
pNK	Peripheral Natural Killer cells
EOPE	Early-Onset Preeclampsia
LOPE	Late-Onset Preeclampsia
GM-CSF	Granulocyte-Macrophage Colony-Stimulating Factor
TNF-α	Tumor Necrosis Factor alpha
VEGF	Vascular Endothelial Growth Factor
IGF	Insulin-like Growth Factor
HLA-DR	Human Leukocyte Antigen-DR isotype
KAR/KIR	Killer Activating/Inhibitory Receptors
sFlt-1	Soluble Fms-like tyrosine kinase-1
VEGFR2	Vascular Endothelial Growth Factor Receptor 2
CI	Confidence Intervals
mAb	Monoclonal Antibodies
BD	Becton Dickinson
FACS	Fluorescence-Activated Cell Sorting
